# The relationship between temperament with nutritional status and anthropometric measurements in adult individuals

**DOI:** 10.1017/jns.2024.89

**Published:** 2025-01-02

**Authors:** Mehmet Arif Icer, Elif Çelik, Aybike Gizem Köse, Makbule Gezmen-Karadag

**Affiliations:** 1 Department of Nutrition and Dietetics, Faculty of Health Sciences, Amasya University, Amasya, Turkey; 2 Department of Nutrition and Dietetics, Faculty of Health Sciences, Suleyman Demirel University, Isparta, Turkey; 3 Department of Nutrition and Dietetics, Faculty of Health Sciences, Gazi University, Ankara, Turkey

**Keywords:** Body mass index, Nine types of temperament, Nutritional status, Temperament types, CHO, Carbohydrate, NTTM, Nine Types of Temperament Model, BMI, Body Mass Index, WHO, World Health Organization, BeBiS, Nutrition Information System, SD, Standard Deviations, PUFA, Polyunsaturated Fatty Acid, MUFA, Monounsaturated Fatty Acid

## Abstract

Interest in studies examining the effect of temperament types on nutrition has recently increased. The aim of this study was to evaluate the relationship between nine types of temperament, anthropometric measurements, and nutrition in adults. This study was conducted on 1317 individuals aged between 18 and 55 years. Descriptive information, dietary habits and anthropometric measurements of the participants were questioned. The Nine Types of Temperament Scale was administered to the individuals and food consumption records were obtained with a 24-hour retrospective reminder method. Type 2 scores of obese participants were higher than those of underweight and normal body weight; Type 8 scores of overweight participants were higher than those of normal body weight. Daily dietary intake of protein, riboflavin, folate, vitamins K, C, calcium, iron, and cholesterol were negatively associated with Type 1 score; protein, magnesium, iron, zinc intake, and water consumption were negatively associated with Type 2 score. Type 3 score was negatively associated with dietary CHO (%), dietary magnesium, iron, and zinc intake and positively associated with water consumption. The results of the study indicate significant relationships between temperament types, dietary habits, and anthropometric measures. In this context, considering temperament types when planning dietary patterns of individuals may be a new approach.

## Introduction

Temperament is defined as the structural integrity of the individual, which is shaped in early childhood and undergoes relatively little change.^(1)^ A personality component present from early infancy, temperament has a biological background and is partly genetically determined. It is more stable and immutable than other personality traits.^(2)^ Temperament traits lead each individual to different motivations and create different behaviours in individuals with these motivations. Different temperament types can explain this situation. Temperament types are the stable characteristics of an individual that make it possible for them to react similarly in every situation that constitutes their behaviour and attitudes.^(3)^

One of these scales, The Nine Types of Temperament Model (NTTM), associates individual differences that enable the perception of human behaviours, actions, and attitudes with temperament. In this model, individuals are thought to be born with one of nine different temperament types.^(3,4)^ While the individual shows other behaviours, the basic temperament is essential. The temperament types defined in the nine types of temperament models are named after the most basic needs and pursuits of individuals belonging to that type. These are: 1. Temperament Type Seeking Perfection, 2. Temperament Type Seeking to Feel Emotions, 3. Temperament Type Seeking Admirable Self-Image, 4. Temperament Type Seeking Meaning of Emotions, 5. Temperament Type Seeking the Meaning of Knowledge, 6. Temperament Type Seeking Intellectual Serenity, 7. Temperament Type Seeking the Joy of Discovery, 8. Temperament Type Seeking Absolute Power, 9. Temperament Type Seeking Sensory Movement Comfort.^(3)^

Previous studies have focused on the role of specific temperamental traits in dieting outcomes. Temperament elements that have been specifically suggested to have potential effects on eating behaviours include impulsivity,^(5,6)^ extraversion,^(7)^ negative emotionality, and self-regulation or effortful control.^(8,9)^ Rothbart *et al.* conceptualised temperament into two main reactivity styles: negative emotionality and boldness/extroversion. Both negative emotionality and boldness are characterised by traits that may increase the consumption of energy-dense, nutrient-poor foods.^(10)^

When the studies investigating the relationship between temperament and body mass index (BMI) in the literature are examined, it is seen that two temperament traits, agility and resilience, partially mediate the relationship between gender and BMI.^(11,12)^ Two studies with children^(13)^ and adults^(14)^ found that certain temperament traits, such as high emotionality and novelty seeking, were associated with higher BMI. Sutin *et al.* showed that high neuroticism is associated with higher BMI, while conscientiousness, extraversion, and openness are protectiveness.^(15–17)^ In these studies, it is seen that different scales have been used to determine temperament types.^(5–7,18)^

Despite the increasing interest in the role of temperament, studies examining the relationship between the nine types of temperament scale, anthropometric measurements and nutritional status in adults are limited. This study aimed to investigate the relationships between temperament types determined using the nine types of temperament model, nutritional status, and anthropometric measurements in adults.

## Methods

### Work plan and participants

This cross-sectional study was conducted with 1317 volunteer adults (673 women and 644 men) aged between 18 and 55. In determining the number of people included in the study, the study of Braet *et al.* ^(5)^ was taken as reference. As a result of the power analysis, it was determined that at least 766 individuals were required to participate in the study in order to have a Type I error (a) = 0.05, effect size 0.24, and power 95%.

Care was taken to ensure that the individuals participating in the study were homogeneously distributed in terms of gender. Individuals younger than 18 and older than 55 years of age, illiterate, pregnant, and lactating women were excluded from the study. This study protocol was approved by the Gazi University Ethics Commission at its meeting dated 13.09.2022 and numbered 15. At the start of questionnaire, full review was obtained and written consent of participants was obtained.

The research data were obtained by the face-to-face questionnaire method. The questionnaire contained information about the participants’ demographic characteristics (age, gender, educational status, place of residence, number of siblings, disease status, use of supplements), anthropometric measurements, dietary habits, and the Nine Types of Temperament Scale. In addition, food consumption records were obtained from the participants with a 24-hour retrospective reminder method.

### Anthropometric measurements

Participants’ body weight and height were taken on a self-reported basis. Body mass index values were calculated as body weight divided by the square of height in metres. Body mass index values were classified according to the World Health Organization’s (WHO) classification. Accordingly, those below 18.5 kg/m^2^ were categorised as underweight, 18.5–24.9 kg/m² as normal weight, 25.0–29.9 kg/m² as overweight, and 30.0 kg/m² and above as obese.^(19)^

### The nine types of temperament scale

The Nine Types of Temperament Scale developed by Yılmaz *et al.* (2014) is a self-report scale for measuring the characteristics of temperament types.^(3)^ The scale consists of 91 items on nine subscales representing nine different temperament types. Participants were asked to respond using a 3-point Likert scale (2 = yes, 1 = sometimes, 0 = no). Higher scores from a temperament type indicate that the participant has more traits related to that temperament type. Cronbach’s alpha value for the total scale was calculated as 0.75.^(3)^ The question numbers indicating the temperament types in the scale are given in Table 1.

**Table 1.** Evaluation of the nine types of temperament scale questions

Types of temperament		Question number	Number of questions
TYPE1	Temperament Type Seeking Perfection	1, 7, 21, 27, 45, 58, 69, 78, 86, 90	10
TYPE2	Temperament Type Seeking to Feel Emotions	11, 19, 33, 41, 44, 49, 53, 61, 70, 83	10
TYPE3	Temperament Type Seeking Admirable Self-Image	12, 18, 26, 35, 38, 59, 65, 87, 88	9
TYPE4	Temperament Type Seeking the Meaning of Emotions	3, 8, 15, 20, 23, 29, 51, 54, 73, 79	10
TYPE5	Temperament Type Seeking the Meaning of Knowledge	2, 9, 13, 34, 39, 47, 57, 60, 66, 71, 76, 81	12
TYPE6	Temperament Type Seeking Intellectual Serenity	4, 6, 28, 36, 48, 55, 64, 68, 82, 84	10
TYPE7	Temperament Type Seeking the Pleasure of Discovery	5, 14, 22, 32, 40, 46, 52, 56, 62, 72	10
TYPE8	Absolute Power Seeking Temperament Type	16, 24, 37, 42, 43, 50, 63, 67, 75, 80	10
TYPE9	Sensory Movement Comfort Seeking Temperament Type	10, 17, 25, 30, 31, 74, 77, 85, 89, 91	10

### Assessment of nutritional status

To determine the energy and nutrient intake of the participants with the daily diet, 24-hour retrospective diet records were taken by the researchers. “Standard Food Recipes for Institutions Serving Mass Nutrition” and “Examples from Turkish Cuisine” were used to determine the amounts of food and the amounts in the portions of the meals.^(16,17)^ The average energy and nutrient values of the foods consumed daily were calculated using the Nutrition Information System (BeBiS) software version 9.0.^(20)^

### Statistical analysis

SPSS 25.0 program was used for data analysis. The chi-square test was used to compare categorical data. Independent Samples *t*-test was used to compare the means ( 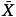 ) and standard deviations (SD) of qualitative data obtained from two groups with normal distribution; the One-Way ANOVA test was used to evaluate the qualitative data of more than two groups. The relationship between two values was analysed using the Pearson correlation test if the data were normally distributed and the Spearman correlation test if not. The limit for statistical significance was set as P < 0.05.

## Results

This study was conducted to determine the relationship between nine types of temperament and nutritional behaviours and anthropometric measurements in adults. Table 2 shows the demographic characteristics of the participants according to gender. The mean age of the individuals was 25.4 ± 7.2 years, and it was found that male participants were older than females (P < 0.05).

**Table 2.** General information and sociodemographic characteristics of participants by gender

	Women (n = 673)	Men (n = 644)	Total (n = 1317)	x^2^	P
	n (%)	n (%)	n (%)		
Age group					
18–30 years	589 (87.5)	515 (80.0)	1104 (83.8)	13.84	**<0.001**
31–55 years	84 (12.5)	129 (20.0)	213 (16.2)		
Age ( 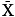 ± SD)	24.5 ± 6.6	26.4 ± 7.6	25.4 ± 7.2	df = 1315 **<0.001**	
Education Status					
Primary/Secondary School	14 (2.0)	16 (2.5)	30 (2.3)	13.48	**0.009**
High School	59 (8.8)	93 (14.4)	152 (11.5)		
University	570 (84.7)	496 (77.0)	1066 (80.9)		
Master’s Degree/PhD	30 (4.5)	39 (6.1)	69 (5.2)		
Marital status					
Married	89 (13.2)	149 (23.1)	238 (18.1)	23.20	**<0.001**
Single	583 (86.6)	492 (76.4)	1075 (81.6)		
Profession					
Student	461 (68.5)	341 (53.0)	802 (60.9)	92.13	**<0.001**
Officer	65 (9.7)	105 (16.3)	170 (12.9)		
Worker	22 (3.3)	72 (11.2)	94 (7.1)		
Self-employment	65 (9.7)	94 (14.6)	159 (12.1)		
Housewife	36 (5.3)	–	36 (2.7)		
Other	24 (3.6)	32 (5.0)	56 (4.3)		
Place of living					
Urban	621 (92.3)	617 (95.8)	1238 (94.0)	7.29	**0.07**
Rural	52 (7.7)	27 (4.2)	79 (6.0)		
Number of siblings					
None	60 (8.9)	56 (8.7)	116 (8.8)	1.04	0.90
One	220 (32.7)	208 (32.2)	428 (32.5)		
Two	195 (29.0)	191 (29.7)	386 (29.3)		
Three and more	198 (29.4)	189 (29.3)	387 (29.4)		
Presence of diagnosed disease					
Yes	62 (9.2)	52 (8.1)	114 (8.7)	0.54	0.46
No	611 (90.8)	592 (91.9)	1203 (91.3)		
Chronic disease					
Cardiovascular diseases	6 (9.7)	8 (15.4)	14 (12.3)	0.86	0.36
Diabetes	3 (4.8)	9 (17.3)	12 (10.5)	4.70	**0.03**
Hypertension	2 (3,2)	10 (19.2)	12 (10.5)	7.69	**0.006**
Digestive system diseases	7 (11.3)	8 (15.4)	15 (13.2)	0.42	0.52
Respiratory system diseases	8 (12.9)	5 (9.6)	13 (11.4)	0.30	0.58
Mental disorders	6 (9.7)	5 (9.6)	11 (9.6)	<0.001	0.99
Musculoskeletal system diseases	9 (14.5)	9 (17.3)	18 (15.8)	0.17	0.68
Endocrine diseases	8 (12.9)	3 (5.8)	11 (9.6)	1.65	0.19
Vitamin/Mineral deficiencies	15 (24.2)	5 (9.6)	20 (17.5)	4.15	**0.04**
Neurological diseases	9 (14.5)	–	9 (7.9)	8.19	**0.004**
Vitamin/mineral supplements					
Yes	94 (14.0)	62 (9.6)	156 (11.8)	5.94	**0.02**
No	579 (86.0)	582 (90.4)	1161 (88.2)		

Table 3 shows the relationship between the participants’ dietary behaviours according to the NTTM. A statistically significant relationship was found between the number of main meals and the Type 9 score. It was found that the frequency of consuming three main meals was higher than the frequency of consuming one main meal in individuals with high Type 9 scores (P < 0.05). There was a relationship between the number of snacks and Type 4, Type 5, and Type 8 scores. The frequency of not consuming snacks and consuming three snacks of individuals with higher Type 4 scores was higher than that of consuming two snacks; the frequency of consuming three snacks of participants with higher Type 4 scores was higher than that of consuming one snack (P < 0.05). Individuals with higher Type 5 and Type 8 scores had a higher frequency of not consuming snacks than the frequency of consuming one or two snacks (P < 0.05). Participants with high Type 1, Type 3, Type 4, Type 7, and Type 8 scores had higher rates of smoking, while participants with high Type 9 scores had lower rates of smoking (P < 0.05). Participants with higher Type 3, Type 4, Type 7, and Type 8 scores had higher rates of alcoholic beverage consumption, while participants with higher Type 9 scores had lower rates of alcoholic beverage consumption (P < 0.05). Considering the amount of alcoholic beverages consumed, individuals who consumed 1001–3000 ml of alcoholic beverages had higher Type 7 scores compared to those who consumed <1000 ml (P < 0.05). Participants who ate with friends had higher Type 1 scores than those who ate alone or with family (P < 0.05). Participants who ate alone had higher Type 3 and Type 5 scores than other groups, and participants who ate alone had higher Type 4 and Type 6 scores than those who ate with family (P < 0.05). Type 8 scores of the participants who ate with their families were higher than the other groups; Type 9 scores of the participants who ate with their families were higher than those who ate alone (P < 0.05).

**Table 3.** Evaluation of participants’ dietary habits and smoking status according to temperament types

Nutrition Behaviours	Type 1	Type 2	Type 3	Type 4	Type 5	Type 6	Type 7	Type 8	Type 9
Number of main meals									
One	19.5 ± 3.9	11.4 ± 4.3	9.1 ± 3.6	10.7 ± 4.2	10.8 ± 5.4	13.1 ± 3.7	10.2 ± 3.1	8.8 ± 4.4	9.8 ± 4.8^a^
Two	18.8 ± 3.9	12.0 ± 4.2	9.5 ± 3.6	9.8 ± 4.3	10.7 ± 5.1	12.7 ± 3.9	10.5 ± 3.5	8.9 ± 4.6	11.2 ± 3.9
Three	18.4 ± 4.0	11.7 ± 4.1	9.5 ± 3.4	9.7 ± 4.2	10.9 ± 4.8	12.6 ± 3.8	10.7 ± 3.5	9.3 ± 4.60	11.6 ± 3.7^a^
P	0.07	0.27	0.75	0.43	0.54	0.22	0.45	0.43	**<0.01**
Number of snacks									
None	19.0 ± 3.2	11.6 ± 2.9	10.3 ± 2.8	10.7 ± 3.4^a^	12.8 ± 4.2^a.b^	11.7 ± 3.3	11.2 ± 2.6	10.7 ± 4.0^a.b^	11.8 ± 2.8
One	18.5 ± 4.0	11.7 ± 4.1	9.5 ± 3.4	9.7 ± 4.4^b^	10.4 ± 5.0^a^	12.6 ± 3.8	10.6 ± 3.5	9.1 ± 4.6^a^	11.4 ± 4.1
Two	18.8 ± 4.1	11.9 ± 4.3	9.3 ± 3.6	9.6 ± 4.2^a.c^	10.8 ± 5.0^b^	12.7 ± 3.9	10.4 ± 3.5	8.7 ± 4.8^b^	11.2 ± 3.9
Three	18.3 ± 3.8	11.8 ± 4.2	9.5 ± 3.4	10.7 ± 4.2^b.c^	11.4 ± 4.6	12.4 ± 3.5	11.0 ± 3.4	9.6 ± 4.2	11.5 ± 3.7
P	0.38	0.86	0.11	**<0.01**	**<0.01**	0.15	0.06	**<0.01**	0.46
Smoking									
Yes	18.9 ± 4.1	12.1 ± 4.4	9.8 ± 3.5	10.3 ± 4.1	10.6 ± 5.1	12.3 ± 3.9	11.1 ± 3.3	10.1 ± 4.5	10.9 ± 3.9
No	18.5 ± 3.9	11.7 ± 4.1	9.4 ± 3.4	9.6 ± 4.3	10.9 ± 4.9	12.7 ± 3.8	10.4 ± 3.5	8.8 ± 4.6	11.6 ± 3.8
P	**0.05**	0.09	**0.03**	**0.01**	0.24	0.11	**<0.01**	**<0.01**	**<0.01**
Number of cigarettes smoked per day									
1–10	18.9 ± 3.9	12.3 ± 4.3	9.9 ± 3.4	10.2 ± 4.0	10.5 ± 4.8	12.2 ± 3.8	10.9 ± 3.3	9.7 ± 4.3	11.1 ± 3.9
11–20	19.0 ± 4.2	12.0 ± 4.4	9.6 ± 3.6	10.5 ± 4.3	10.6 ± 5.3	12.5 ± 4.1	11.4 ± 3.2	10.4 ± 4.7	10.8 ± 3.8
>20	19.1 ± 4.9	11.2 ± 4.8	11.5 ± 3.8	9.5 ± 3.8	10.5 ± 5.7	11.4 ± 3.8	10.9 ± 3.4	12.1 ± 5.0	9.4 ± 3.8
P	0.99	0.10	0.24	0.73	0.16	0.76	0.08	0.12	0.25
Consumption of alcoholic beverages									
Yes	18.8 ± 4.4	11.9 ± 4.7	10.1 ± 3.6	10.5 ± 4.1	10.5 ± 4.9	12.7 ± 3.9	11.4 ± 3.5	9.7 ± 5.0	10.9 ± 4.0
No	18.6 ± 3.9	11.8 ± 4.0	9.4 ± 3.4	9.6 ± 4.2	10.9 ± 4.9	12.5 ± 3.8	10.5 ± 3.4	9.0 ± 4.5	11.5 ± 3.8
P	0.46	0.63	**<0.01**	**0.01**	0.25	0.60	**<0.01**	**0.04**	**0.05**
Amount of alcoholic beverages consumed									
0–1000 ml	18.8 ± 4.5	12.0 ± 4.7	9.9 ± 3.5	10.4 ± 4.2	10.6 ± 5.1	12.9 ± 3.9	11.1 ± 3.6^a^	9.5 ± 5.0	11.1 ± 3.9
1001–3000 ml	19.5 ± 4.1	11.7 ± 4.7	10.8 ± 4.0	10.9 ± 3.5	9.3 ± 5.9	11.9 ± 3.8	12.7 ± 2.7^a^	10.8 ± 5.1	9.5 ± 4.5
>3000 ml	16.0 ± 4.2	9.9 ± 4.0	12.3 ± 4.6	9.5 ± 5.1	11.6 ± 6.3	10.8 ± 5.1	12.8 ± 3.6	12.5 ± 3.4	11.9 ± 3.4
P	0.14	0.43	0.11	0.70	0.42	0.19	**0.05**	0.13	0.13
The person with whom you eat									
Family	18.4 ± 3.8^a^	11.9 ± 3.9	9.4 ± 3.3^a^	9.6 ± 4.4^a^	10.9 ± 4.8^a.b^	12.2 ± 3.9^a^	10.5 ± 3.4	9.5 ± 4.4^a^	11.6 ± 3.7^a^
Lonely	18.3 ± 4.3^b^	11.7 ± 4.5	10.1 ± 3.6^a.b^	10.5 ± 4.1^a^	11.8 ± 5.1^a.c^	13.2 ± 3.6^a^	10.8 ± 3.7	9.4 ± 5.2^b^	10.8 ± 4.3^a^
Friends	19.3 ± 4.1^a.b^	11.8 ± 4.4	9.3 ± 3.6^b^	9.8 ± 4.2	10.1 ± 4.9^b.c^	12.8 ± 3.7	10.7 ± 3.4	8.4 ± 4.5^a.b^	11.3 ± 3.8
P	**<0.01**	0.73	**0.01**	**0.01**	**<0.01**	**<0.01**	0.44	**<0.01**	**0.02**

There is a statistically significant difference between the data indicated with the same letter.

The BMI evaluation of the participants according to temperament types is given in Table 4. A statistically significant difference was found between BMI and Type 2, Type 5, and Type 8 scores (P < 0.05). Obese participants had higher Type 2 scores than underweight and normal body weight participants (P < 0.05). Underweight participants had higher Type 5 scores than obese participants (P < 0.05). Overweight participants had higher Type 8 scores than those with normal body weight (P < 0.05).

**Table 4.** Evaluation of BMI of participants according to temperament types

	BMI				
Temperament Types	Underweight (n = 119)	Normal (n = 807)	Overweight (n = 319)	Obese (n = 72)	P
TYPE1	18.7 ± 3.4	18.7 ± 4.1	14.5 ± 3.9	18.4 ± 3.8	0.73
TYPE2	11.6 ± 3.9^a^	11.7 ± 4.1^b^	11.9 ± 4.4	13.2 ± 4.1^a.b^	**0.02**
TYPE3	9.7 ± 3.3	9.4 ± 3.4	9.6 ± 3.7	9.2 ± 3.5	0.65
TYPE4	10.8 ± 4.7	9.7 ± 4.1	9.8 ± 4.3	9.3 ± 4.2	0.06
TYPE5	11.7 ± 4.5^a^	10.7 ± 4.9	11.1 ± 4.9	9.8 ± 5.2^a^	**0.03**
TYPE6	12.8 ± 3.6	12.7 ± 3.8	12.3 ± 3.8	11.8 ± 4.1	0.12
TYPE7	10.6 ± 3.3	10.6 ± 3.50	10.5 ± 3.4	11.1 ± 3.4	0.60
TYPE8	9.3 ± 4.7	8.7 ± 4.6^a^	9.9 ± 4.4^a^	10.1 ± 4.9	**<0.001**
TYPE9	11.2 ± 3.4	11.1 ± 3.9	11.6 ± 3.8	12.2 ± 4.0	0,14

BMI, Body Mass Index: a statistically significant difference exists between the data shown with the same letter.

**Fig. 1.**
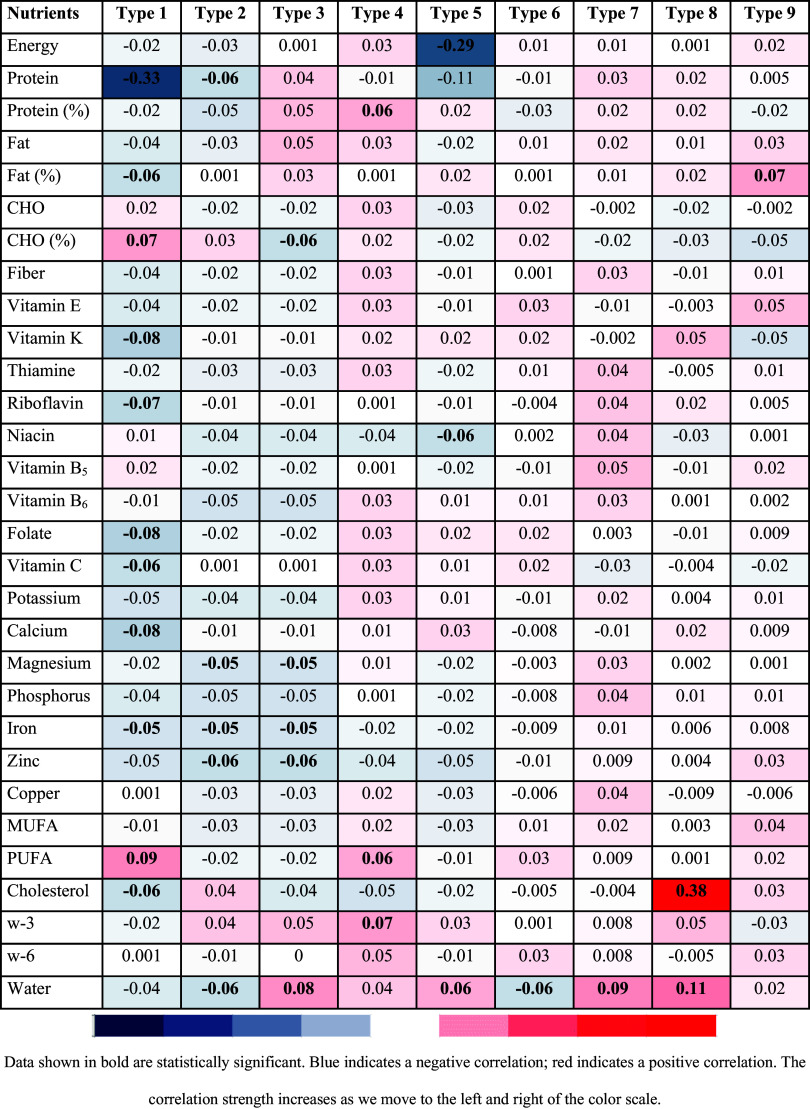
The relationship between participants’ temperament types and macro and micronutrient intakes.

The correlation between temperament types and nutrients is given in Fig. 1. There was a negative and statistically significant correlation between daily dietary protein, daily fat intake percentage, riboflavin, folate, vitamins K and C, calcium, iron, cholesterol intake, and Type 1 score, and a positive correlation between dietary CHO (%) and dietary polyunsaturated fatty acid (PUFA) and Type 1 score (P < 0.05). Type 2 score was negatively correlated with daily dietary protein, magnesium, iron, zinc intake, and water consumption (P < 0.05). Type 3 score was negatively correlated with dietary CHO (%), dietary magnesium, iron, and zinc intake, and positively correlated with water consumption (P < 0.05). There was a positive correlation between daily dietary protein, PUFA, and w-3 fatty acid intake and Type 4 score (P < 0.05). Type 5 score was negatively correlated with daily dietary energy and niacin intake and positively correlated with water consumption (P < 0.05). There was a negative correlation between water consumption and Type 6 score and a positive correlation between Type 7 and Type 8 scores (P < 0.05). There was a positive correlation between daily dietary cholesterol intake and Type 8 score (P < 0.05). There was a positive correlation between daily dietary fat consumption percentage and Type 9 (P < 0.05).

## Discussion

This study examined the relationship between temperament types determined by the nine types of temperament model and anthropometric measurements and dietary habits. The study results show a relationship between temperament types and anthropometric measurements, some eating habits, and dietary intake of various nutrients. The limited number of studies examining this issue in the literature and the fact that these studies generally focus on negative affect reveals the deficiencies in this field.

Research consistently emphasises the negative impact of skipping meals, especially breakfast, on physical and mental health.^(21–23)^ In one study, neglecting breakfast was associated with reduced energy intake.^(21)^ Skipping meals is positively correlated with mental distress.^(23)^ A study conducted on children reported that children with extroverted and internalising temperaments were likelier to skip breakfast every day. In contrast, children with average sociability scores had regular breakfast habits.^(22)^ This study found that the frequency of consuming three main meals was higher in individuals with high Type 9 scores compared to individuals who consumed one main meal. The study population consisted of adult individuals, and no other study in the literature examines the relationship between temperament types and meal skipping in adults. Therefore, this study is predicted to lead to new studies on whether temperament types are effective in meal skipping.

Research shows that smoking and alcohol consumption are associated with poor eating habits, such as low intake of fruits, vegetables, and cereals and high fat and alcohol consumption.^(24–26)^ These habits can lead to lower nutrient intake and poorer food choices, negatively affecting overall health and nutrition. It has been reported that smokers tend to have lower fear and control and drive higher anger and unstable emotional temperament.^(27)^ Another study supported this by showing that smokers have lower harm avoidance and higher novelty-seeking, reward dependence, and persistence.^(28)^ Participants with higher Type 1, Type 3, Type 4, Type 7, and Type 8 scores had higher rates of smoking, while participants with higher Type 9 scores had lower rates of smoking (P < 0.05). Participants with higher Type 3, Type 4, Type 7, and Type 8 scores had higher rates of alcoholic beverage consumption, while participants with higher Type 9 scores had lower rates of alcoholic beverage consumption. In line with the results obtained from the literature and this study, it can be concluded that temperament types may be effective on smoking and alcohol use habits. However, there is a need for more comprehensive studies using similar temperament scales to reveal more clearly which temperament types affect these habits.

Although there has been a recent increase in studies investigating the effects of temperament types on the risk of obesity development, these studies have often focused on children.^(9,29)^ In adults, neurotic personality trait is associated with higher body mass index.^(30,31)^ In contrast, people with self-regulation and conscientiousness temperaments are reported to have a lower risk of developing obesity.^(9,31)^ van Eeden *et al.* (2020) found that higher negative affect and lower effortful control in pre-adolescence were generally associated with higher BMI in young adulthood. The same study predicted that common effortful control may consistently increase the development of obesity in adolescents.^(29)^ In this study, it was observed that obese individuals had higher scores in the temperament type seeking to feel emotions (Type 2) and absolute power-seeking temperament type (Type 8) and lower scores in the temperament type seeking the meaning of information (Type 5). This result suggests that individuals predisposed to Type 2 and Type 8 temperament types may be at higher risk for obesity, but more studies are needed in this regard.

Negative affect temperament type is generally associated with consuming more energy-dense, nutrient-poor foods, high-sugar foods, and sugar-sweetened beverages. Kidwell *et al.* (2023) found that adolescents with high negative affectivity were more likely to consume foods and beverages and sugar than those with low negative affectivity. Another result of that study was that having internalising (anxious and dependent) and externalising (hyperactive and aggressive) temperaments in 18-month-olds is a risk factor for greater consumption of sweet drinks and foods at ages three and seven.^(32)^ Another study showed that active and social temperament in 18-month-old infants was associated with higher daily fruit and vegetable consumption at three and seven years of age.^(7)^ In the present study, Type 2 score was negatively correlated with daily dietary protein, magnesium, iron, zinc intake, and water consumption. There was a positive correlation between daily dietary protein, PUFA, and w-3 fatty acid intake and Type 4 score. Another study result was a negative correlation between the score of perfection-seeking temperament type (Type 1) and daily dietary intake of protein and protein, vitamin K, riboflavin, folate, vitamin C, calcium, iron, and cholesterol. In light of the present data, it can be considered that predisposition to some temperament types may affect nutritional status and dietary intake levels of some macro/micronutrients and bring potential health risks, especially obesity.

This study has some limitations. The Nine Types of Temperament Scale used in this study has yet to be used before to assess the relationship between temperament and nutritional status in adults. Although this adds a unique value to the study, it needs to be clearer to compare the results of the survey with the results of other studies using different scales to determine temperament types.

## Conclusions

The results obtained from this cross-sectional study show that some temperament types in the nine types of temperament models are associated with anthropometric measurements and nutritional behaviours. When it is considered that genetics is influential in determining temperament and does not change frequently, it is essential to design interventions by considering temperament characteristics instead of only providing nutritional recommendations to individuals. With the new studies to be conducted, it can be thought that determining the diet according to temperament types may be a new alternative strategy to improve the nutritional status and prevent the development of obesity by revealing the effects of temperament types on nutritional status more clearly. Our study anticipates accelerating new studies to be planned for this purpose.
